# Simple Measures of Function and Symptoms in Hospitalized Heart Failure Patients Predict Short-Term Cardiac Event-Free Survival

**DOI:** 10.1155/2014/815984

**Published:** 2014-02-09

**Authors:** Evanthia Zaharias, Janine Cataldo, Lynda Mackin, Jill Howie-Esquivel

**Affiliations:** ^1^Department of Case Management, University of California, San Francisco Medical Center, 505 Parnassus Avenue, San Francisco, CA 94143-0208, USA; ^2^Department of Physiological Nursing, University of California, San Francisco, 2 Koret Way, San Francisco, CA 94143-0610, USA; ^3^Department of Physiological Nursing, University of California, 2 Koret Way, San Francisco, CA 94143-0610, USA

## Abstract

*Background*. Heart failure (HF) is a prevalent chronic condition where patients experience numerous uncomfortable symptoms, low functional status, and high mortality rates. *Objective*. To determine whether function and/or symptoms predict cardiac event-free survival in hospitalized HF patients within 90 days of hospital discharge. *Methods*. Inpatients (*N* = 32) had HF symptoms assessed with 4 yes/no questions. Function was determined with NYHA Classification, Katz Index of Activities of Daily Living (ADLs), and directly with the short physical performance battery (SPPB). Survival was analyzed with time to the first postdischarge cardiac event with events defined as cardiac rehospitalization, heart transplantation, or death. *Results*. Mean age was 58.2 ± 13.6 years. Patient reported ADL function was nearly independent (5.6 ± 1.1) while direct measure (SPPB) showed moderate functional limitation (6.4 ± 3.1). Within 90 days, 40.6% patients had a cardiac event. At discharge, each increase in NYHA Classification was associated with a 3.4-fold higher risk of cardiac events (95% CI 1.4–8.5). Patients reporting symptoms of dyspnea, fatigue, and orthopnea before discharge had a 4.0-fold, 9.7-fold, and 12.8-fold, respectively, greater risk of cardiac events (95% CI 1.2–13.2; 1.2–75.1; 1.7–99.7). *Conclusions*. Simple assessments of function and symptoms easily performed at discharge may predict short-term cardiac outcomes in hospitalized HF patients.

## 1. Introduction

Heart failure (HF) is a complex syndrome resulting from structural or functional disorders of the heart that impair ventricular ability to fill with or eject blood [1]. As the final stage of many types of heart disease, HF is a prevalent chronic condition and a major public health issue [2]. The costs of HF are large: it has been called the “most costly cardiovascular disorder” in the USA [3]. The total healthcare expenditure for HF in the USA was 34.4 billion dollars in 2010 [4], and hospitalizations are the top contributor to these costs [2]. HF is the main reason for 6.5 million hospital days annually [1] and the most common condition for hospital admission in people aged 65 and over [2]. The hospital burden of HF is expected to increase with the rapid aging of the USA population: 72 million adults are projected to be over age of 65 by 2030 [5]. Costs will further increase since HF is part of a key quality-related provision in the Affordable Care Act of 2010. This provision decreases hospital reimbursements for 30-day readmission rates not meeting targets for multiple chronic conditions that include HF [6].

Despite current medical treatment, the prognosis for HF is poor, with a 5-year mortality rate of 45–60% [2]. In addition to high mortality, disability levels in HF patients are also consistently high. A recent analysis in a national community-based sample of patients who reported having HF between 2003 and 2008 found that 11% had disability in activities of daily living (ADLs) and 57% had mobility disability (much difficulty with or inability to walk 2-3 blocks or climb 10 steps). These rates were constant among HF patients in study cohorts back to 1988 [7].

The connection between HF and disability can be understood by the process described in Nagi's model of disability, in which the pathology of a condition leads first to physical signs and symptoms and impairments in body systems and then to functional limitations (physical, psychological, and/or social), and finally to disability [8]. Various measures of physical function have been shown to predict rehospitalization and survival in patients with HF. Decreased mobility in hospitalized patients is associated with adverse outcomes like functional decline [9, 10], new institutionalization, and death [9].

Functional decline is both preceded and made worse by signs and symptoms of a condition like HF, according to Nagi's model [8]. HF patients commonly experience a highly variable symptom burden and diminished health-related quality of life [11–13]. The symptom burden of HF, including shortness of breath, fatigue, lower extremity edema, and orthopnea [1] is similar to that of advanced cancer [14].

Few researchers have studied whether HF symptoms in an inpatient setting predict postdischarge outpatient outcomes like rehospitalization and mortality. One large retrospective study examined charts of patients hospitalized with decompensated HF and found that those with fewer documented in-hospital symptoms had worse short-term (30 days) mortality [15]. Another prospective cohort study identified symptom clusters in hospitalized HF patients, termed “dyspneic” and “weary,” and found that higher levels of distress from these clusters independently predicted one-year cardiac death-free survival and cardiac rehospitalization-free survival, respectively [16]. Although it is known that the timing of assessment of potential risk factors (e.g., symptoms) during the illness trajectory may affect patient outcomes [17], to our knowledge, no studies have examined whether the timing of HF symptom measurement during hospitalization (e.g., at admission or discharge) might predict outcomes such as rehospitalization or survival.

Predictors that help to identify hospitalized HF patients who are at higher risk of adverse cardiac outcomes allow for the development of more effective in-hospital care plans and more targeted interventions to prevent future cardiac events or less aggressive curative efforts if indicated, along with improved discharge coordination and follow-up care. This pilot study was part of a parent study to characterize levels of mobility in hospitalized HF patients using accelerometers. The purpose of this study was to explore multiple measures of function as well as symptoms and to examine related factors that might predict short-term cardiac event-free survival.

### 1.1. Aims

The specific aims of this pilot study were to examine the (1) function of HF patients at home and between two points in time during hospitalization and (2) symptoms of HF patients at two points in time during hospitalization (3) to determine whether function and/or symptoms predict cardiac event-free survival up to 90 days after hospital discharge. Time points were the study start, which was up to 48 hours after hospital admission, and the study end, which was up to 7 days after hospital admission or the day of discharge if the hospital stay was less than 7 days. Function was measured by NYHA Classification, self-report of home exercise, the Katz Index of independence in ADLs, the short physical function battery, the Karnofsky Performance status Scale, and ambulation. Ambulation was defined as the average daily time spent lying, sitting, and standing or walking. Symptoms were measured by yes/no questions regarding shortness of breath, fatigue, orthopnea, and edema. Cardiac events were defined as rehospitalization attributed to a cardiac cause, heart transplantation, or death.

## 2. Materials and Methods

### 2.1. Study Design, Setting, and Sample

A convenience sample of 32 patients aged 30 and above was recruited for this prospective cohort study, which took place on two inpatient telemetry units at a large urban academic hospital. The study was approved by the local Institutional Review Board and inclusion criteria included a primary or secondary diagnosis of HF as determined by medical record review, ability to ambulate with or without an assistive device during the month prior to hospitalization, having a doctors' activity order that allowed the patient to be ambulatory, ability to speak English, and no isolation precautions. Exclusion criteria included dementia as measured by the MiniCog [18] or indicated as severe in the medical record, delirium as measured by the confusion assessment method (CAM) [19], and living in a skilled nursing facility prior to admission.

A trained graduate nursing student conducted all study procedures and data analysis in collaboration with the principal investigator of the parent study. All patients admitted to the study units under either cardiology or medical services within the prior 48 hours were prescreened daily via chart review between April 2010 and February 2011. After prescreening, 103 patients were considered for study approach and 32 were enrolled. Of the 71 patients not enrolled, 46% had a short length of stay (LOS). Short LOS was defined as a discharge planned on the same or next day according to electronic medical record notes, the bedside nurse, or the medical team. Refusals accounted for 27% of those not enrolled. The final 27% were not enrolled for various reasons including having lower extremity skin problems that would interfere with monitor attachment (e.g., leg wraps), speaking a language other than English, cognitive problems, transfer off the unit, or not actually being ambulatory.

Once consented, the Mini-Cog and the CAM were administered to screen for cognitive impairment and delirium, respectively, and the patient was enrolled if eligible. Including the enrollment visit, the study procedure consisted of up to 5 hospital visits and one follow-up phone call after discharge (Table 1).

### 2.2. Data Collection

Demographic information was collected from medical records at enrollment and confirmed by patient interviews. Clinical information including co-morbidities (high risk diagnosis for the elderly score, HRDES [20]) was extracted from medical records when the patient was discharged. Vital signs, NYHA Classification, and symptoms were determined by the study nurse at the beginning and end of the study; NYHA Class was also assessed at home prior to admission (based on self-report of symptoms with activity). Function was assessed at home (self-report of home exercise), at the beginning of the study (Katz Index, KPS, and SPPB), and during the study (ambulation). Both assessments pertaining to the patient at home (NYHA Class and home exercise) were self-reported at the study start in the hospital, based on the patient's recall of his or her functioning prior to admission. The Katz Index and the KPS were nurse-administered questionnaires, while the SPPB was a direct performance measure. Ambulation was measured continuously during the study period via wireless accelerometer monitors.

Telephone follow-up occurred at 90 days following discharge to determine whether patients experienced hospitalizations or emergency department visits. In addition, they were asked about which symptoms led them to seek care. Deaths were confirmed by public death records including internet-accessible obituaries and the Social Security Death Index [21].

### 2.3. Measures

Refer to Table 2 for a summary of basic information about study measures.

#### 2.3.1. Function

*NYHA Classification.* It was originally developed in 1928 [22]. This well-established instrument describes functional status that ranges from Class I for patients who have no symptoms to Class IV for patients who are symptomatic at rest [23, 24]. Since it indicates what level of physical activity provokes HF symptoms, NYHA Classification can be viewed as a combined assessment of physical function and symptoms. Classification by NYHA is inherently subjective since it depends on a healthcare provider's interpretation of various levels of physical activity (e.g., what is meant by “ordinary” activity) and whether limitations should be called “slight” or “marked” [22]. This subjectivity has caused some to question the validity of NYHA Classification [25]. However, studies show that NYHA Classification is a valid measure. Although based on subjective assessment by the examiner, the NYHA instrument is valid, correlating well with other functional measures including exercise testing [17, 26–28], and tests have found the NYHA classification to be moderately reliable [25, 29].

*The Katz Index of Independence in ADLs.* It measures a person's ability to independently perform ADLs. The Katz Index scores individuals based on six basic ADLs (bathing, dressing, toileting, transferring, continence, and feeding), indicating whether they are dependent (0 points) or independent (1 point) in each function. A score of 6 indicates full function, a score of 4 indicates moderately impaired function, and a score of 2 or less denotes severe functional impairment. Created nearly half a century ago, the Katz Index remains a best-practice tool for assessing functional status in older adults that has been found to be both reliable and valid in studies in multiple populations including older adults and poststroke patients [30, 31].

*The Short Physical Performance Battery (SPPB).* It is a set of three tests that objectively measure lower extremity physical performance. Patients with scores of 10–12 are classified as having minimal limitations, scores of 7–9 have mild limitations, scores of 4–6 suggest moderate limitations, and scores of 0–3 indicate severe performance limitations. The SPPB was originally developed in the 1990s, when it was tested in a large epidemiological study of nondisabled community-dwelling older adults [32, 33]. The SPPB was found to be valid and reliable in this study. Other studies have further shown reliability [34], and validity has been demonstrated through correlation with 400-meter walk tests [35].

*Ambulation.* It was measured via Micro Care Timeliness Monitors, miniature recording 3-axis accelerometer monitors (AugmenTech, Pittsburgh, PA). These monitors were previously tested in a mobility study of hospitalized older adults and showed reliability (internal consistency) and validity (comparability to direct patient observation) [36]. In the present study, two accelerometer monitors were programmed and attached to the thigh and ankle of the patient for up to five days, or until discharge. Monitors were removed daily, skin condition was checked, and a new set of monitors was attached to the ankle and thigh on the opposite leg. The HyperTerminal PE program (Hilgraeve Inc., Monroe, MI) was used to program monitors and download data. Gauze pads were used to cushion the monitors against the skin and Tegaderm (3M, St. Paul, MN) secured the monitors.

*The Karnofsky Performance Status Scale (KPS).* It is a global measurement of function which uses a 100-point rating scale to assess the impact of a health condition on ability to work and care for oneself. The rating scale ranges in increments of 10 from normal function (score of 100) to absence of function (score of 0, patient deceased), and it was originally developed in 1948 to assess function in cancer patients [37]. Reliability has been tested and validity of the KPS has been established with respect to measures of ADL function and quality of life [38, 39].

#### 2.3.2. Symptoms

*Patient Symptoms.* They were assessed via yes/no questions regarding whether or not the patient was experiencing shortness of breath, fatigue, lower extremity edema, or orthopnea.

*Self-Report of Home Exercise.* It was determined by asking patients whether or not they do exercise at home.

### 2.4. Statistical Analysis

Demographic and clinical data were analyzed using SPSS Statistics 19 software (IBM Corp., Armonk, NY). Descriptive statistics were used to summarize demographic and clinical data, including function and symptoms. Accelerometer monitor data was processed using Excel (Microsoft, Redmond, WA) and SPSS Statistics 19. Ambulation, defined as average daily time spent in each position (lying, sitting, and standing or walking), was calculated during the study period for each patient. Full analysis of the ambulation data is presented elsewhere. For this analysis, data on function and symptoms were used as independent variables for survival analysis. Survival analyses were completed using a univariate Cox proportional-hazards regression with time to first cardiac event after discharge as the outcome variable. An alpha of 0.05 was used. Hazard ratios and confidence intervals were calculated to identify predictors of cardiac events.

## 3. Results

### 3.1. Sample Characteristics

Thirty-two patients with a mean age of 58.2 ± 13.6 years participated in the study, and 78.1% were men (Table 3). More than half self-identified as white (59.4%), 31.3% as African-American, and 9.4% as Asian or Pacific Islander. Most had a history of hypertension (71.9%) and mean creatinine on admission was 1.9 ± 1.7 g/dL. Most patients had systolic HF: 70.9% had an EF of less than 40%. The comorbidity score or mean HRDES was 3.3 ± 1.7, and the majority (53.1%) of patients fell into the intermediate category, with a corresponding 31% chance of dying in the next year. Length of hospital stay ranged from 1 to 41 days, with a mean of 9.5 ± 9.9; median stay was 6.5 days. Two patients (6.3%) received an LVAD during the original study admission but after the study was complete; these patients had the longest LOS at 41 days each. Within 90 days after discharge, a total of 3 patients (9.4%) died, 1 patient received a heart transplant, and 11 (34.4%) had cardiac rehospitalization.

### 3.2. Functional Status

The proportion of patients in each NYHA Class differed across time points (Table 4). Close to half (46.9%) of patients reported having experienced symptoms at rest (NYHA Class IV) at home prior to admission. By the start of the study, a median of 1.0 days after admission, 18.8% reported symptoms at rest. Only one patient (3.1%) reported symptoms at rest at the study end, a median of 1.0 days prior to discharge.

Most patients (62.5%) reported exercising at home prior to admission, and the mean Katz Index score was 5.6 ± 1.1 (possible 0–6) indicating near independence in ADLs. The mean SPPB score at the study start was 6.4 ± 3.1, placing the mean just above the classification of moderate physical function limitation. The largest proportion of patients (38.7%) had scores in the moderate functional limitation category (score 4–6). Ambulation measurement during the study showed that patients spent an average of  59 ± 43 minutes daily standing or walking in the hospital and an average of 16.8 ± 3.2 hours lying down.

The mean KPS score was 71.1 ± 9.0, with scores ranging from 50 to 90. Most patients (71.9%) fell into the middle KPS category of 50–79, indicating that they were unable to work but were able to live at home and care for most needs with varying amounts of assistance. No patients were unable to care for themselves and required the equivalent of hospital care (score <50), and 28.1% felt they were able to carry on normal activity without any special care (scores 80–100).

### 3.3. Symptoms

**Figure 1:**
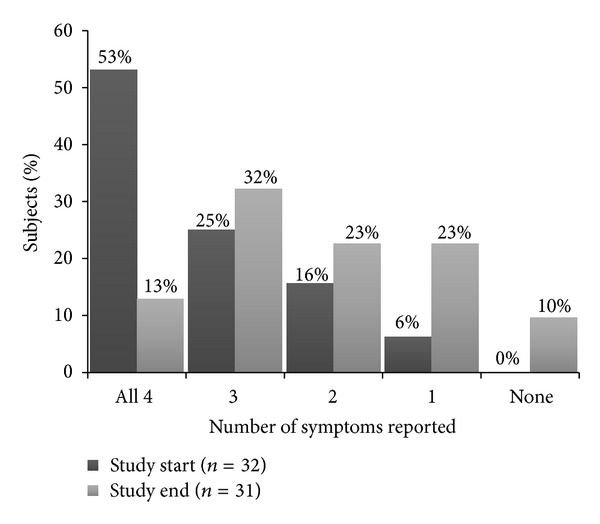
Percent of patients reporting different numbers of symptoms at study start and study end. Study start was 1.0 days (median) after admission; study end was 1.0 days (median) before discharge. Symptoms assessed were shortness of breath, fatigue, orthopnea, and edema.

At the beginning of the study, the median number of symptoms reported was 4.0 out of the four HF symptoms assessed (shortness of breath, fatigue, orthopnea, and edema). The mean was 3.3 ± 1.0 symptoms, and all patients reported at least one symptom (Table 5). At the study end, the median number of symptoms reported decreased to 2.0, the mean was 2.1 ± 1.3 symptoms, and 10% of patients reported no symptoms (Figure 1).

**Figure 2:**
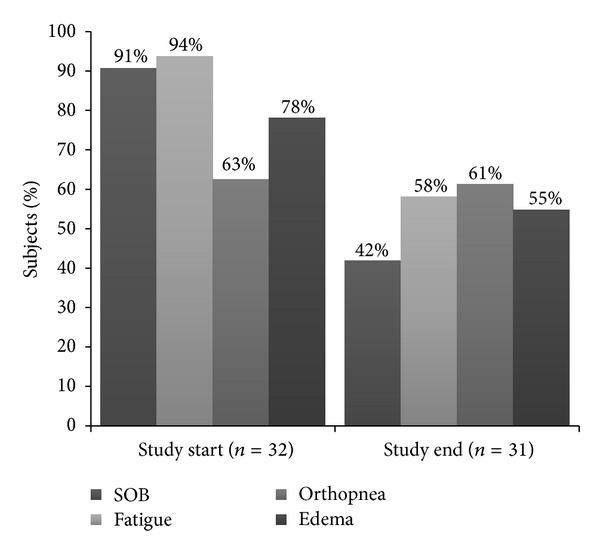
Percent of patients reporting individual symptoms common in HF at the beginning and end of the study. SOB= shortness of breath. Study start was 1.0 days (median) after admission; study end was 1.0 days (median) before discharge.

Fatigue was the most prevalent symptom (Figure 2) at both the study start (94%) and the study end (58%). Prevalence of SOB decreased the most between the study start and end, from 91% to 42%, and orthopnea decreased the least, from 63% to 61%.

### 3.4. Prediction of Cardiac Event-Free Survival

At least one cardiac event occurred in 13 patients (40.6%) within 90 days after discharge. Most initial cardiac events (*n* = 11) were cardiac readmissions, including one patient readmitted after the study for heart transplantation. Implantations of LVADs in two patients were not included as cardiac events since they occurred during the same admission as the study. One patient who died within 90 days had a prior cardiac readmission during the follow-up period, so that readmission was analyzed as the first cardiac event. The two additional deaths were analyzed as the first cardiac events.

Of the demographic and clinical characteristics analyzed, two factors were found to be associated with cardiac event-free survival (Table 6). Results showed that patients were 4.2 times less likely to have a cardiac event if they had a history of hypertension (HR 0.238; 95% CI 0.08–0.71). Additionally, an increased LOS was associated with a higher risk of cardiac events. Results showed an 8.5% increase in risk with each additional day in the hospital (HR 1.085; 95% CI 1.03–1.15).

Among the physical function measures, each increase in NYHA Class at the study end was associated with a 3.4-fold higher risk of cardiac events within 90 days (HR 3.404; 95% CI 1.37–8.46). The sole measure of global function, KPS, was not predictive of cardiac events at the alpha level designated for this study (0.05).

Three of the 4 symptoms reported by patients at the study end (median of 1.0 days before discharge) were significant predictors of cardiac events. Patients reporting SOB at the study end had a 4.0-fold greater risk of cardiac events than those who did not report this symptom (HR 3.962; 95% CI 1.19–13.22). Risk of cardiac events was 9.7 times higher among patients reporting fatigue at the study end (HR 9.661; 95% CI 1.24–75.06), and risk of cardiac events was 12.8 times higher among patients who reported orthopnea at study end (HR 12.807; 95% CI 1.65–99.73). No symptoms reported at study start predicted outcomes, and edema was the only symptom not associated with outcomes at study end.

## 4. Discussion

In this study, we found that higher NYHA Classification and presence of three symptoms (shortness of breath, fatigue, or orthopnea) at the study end (a median of 4.5 days after admission and 1.0 days before discharge) predicted cardiac events in HF patients within 90 days after hospital discharge. These results are unique because they suggest that a quick, simple assessment of function and symptoms that can easily be made at the bedside by physicians or nurses before hospital discharge may be a meaningful way to predict short-term cardiac outcomes in HF patients.

### 4.1. Function

NYHA Classification at study end (median of 1.0 days before discharge) was found to be predictive of cardiac events in this study, and this is consistent with a recent review that examined risk factors for HF hospitalization. The review noted that NYHA Classification at hospital discharge has been shown to predict both 30-day and 1-year readmissions [17]. Other researchers have also shown that NYHA Classification is associated with mortality. Results from an outpatient study by Devroey and Van Casteren showed that patients who died of HF within 6 months of their diagnosis had a higher NYHA Class at diagnosis than those who did not die [40]. Another study of function in older hospitalized HF patients found that increasing preadmission NYHA Class was associated with greater mortality over a follow-up period of over a year [26]. This is in contrast to results reported here, in which only predischarge, not pre-admission, NYHA Classification predicted outcomes.

The distribution of NYHA Class results from the 32 patients changed from home to the study start and to the study end. Thus, the NYHA Class reflects the dynamic clinical course of HF, from the overall severity of HF exacerbation in the study cohort prior to admission to the symptomatic relief obtained after initial treatment (study start) to further improvements after ongoing treatment (study end). It is common to find NYHA Classification in patient's hospital admission notes, but our results suggest that particular attention should also be paid to assessing and documenting NYHA Classification close to hospital discharge for optimal discharge planning.

Chiarantini and colleagues [26] found that SPPB was related to survival in their cohort study involving older HF patients. A higher SPPB score at discharge conferred greater risk of mortality over a follow-up period of just over one year. These results differ from our study, which found no significant relationship between cardiac events (including mortality) and the SPPB. However, our population was younger, the study follow-up period was shorter, and the SPPB was assessed closer to admission, representing a different time point.

### 4.2. Symptoms

Among the three symptoms that predicted outcomes, orthopnea conferred the greatest risk for cardiac events, followed by fatigue and shortness of breath. Edema was not predictive at either of the time points measured. These results are similar to those found by investigators in the large European beta-blocker drug trial, COMET (Carvedilol or Metoprolol European Trial). In a secondary analysis of outpatients, investigators reported the patient's NYHA Class and edema and asked patients about breathlessness, fatigue, angina, and orthopnea [41, 42]. The same assessments were conducted at baseline and up to a follow-up period of nearly five years. Univariate analysis showed that only breathlessness, orthopnea, and fatigue were significantly related to the development of worsening HF and to reduced survival. Assuming that breathlessness is equivalent to shortness of breath, it is striking that these are the same three symptoms found to be significant in univariate analyses in the present study. The COMET analysis [41, 42] differed from our study in that it took place in an outpatient setting and examined long-term outcomes.

Results of our study are congruent with the findings of Song and colleagues [16], who completed a prospective cohort study to identify symptom clusters among inpatients with HF exacerbation and determine their impact on cardiac-related death and rehospitalization. Symptoms were assessed by questionnaire 1-2 days prior to discharge, and monthly followup lasted one year. Two main physical symptom clusters emerged, termed “dyspneic” (shortness of breath, difficulty of breathing when lying flat, and waking up breathless at night) and “weary” (lack of energy, lack of appetite, and difficulty sleeping). Key results from the study were that a higher level of distress from the weary symptom cluster was an independent predictor of cardiac rehospitalization-free survival, and higher distress from the dyspneic symptom cluster was an independent predictor of cardiac death-free survival. These analyses controlled for clinical variables like age, sex, HF etiology, BMI, EF, and comorbidities, making a strong argument that the symptom experience alone is related to negative outcomes. The “dyspneic” cluster includes two of the three symptoms that were significant in the present study, and they were also measured at a similar time close to discharge. This supports our results and suggests that these same symptoms might also predict longer-term outcomes. Both the symptom cluster study [16] and the COMET analysis [41, 42] corroborate our finding that symptoms can predict outcomes in hospitalized HF patients.

### 4.3. Timing

Our results demonstrate that the timing of assessment is of paramount importance when predicting outcomes. It is known that the point in time when a risk factor is measured during the course of illness (e.g., at diagnosis, hospital admission, or discharge) may affect prediction of outcomes [17]. Hospitalization with acute HF is a time of high risk for patients, in which adverse outcomes are more likely [43]. In this study, function at home prior to admission and function and symptoms at the study start were not related to outcomes, whereas both function (NYHA Classification) and symptoms at the study end were found to predict outcomes. By the end of the study, patients had undergone medical therapy for 4.5 days (median time between admission and study end) and they were 1.0 days (median) from discharge. One possibility is that these patients were inadequately diuresed, leading to continued symptoms close to discharge. Another possibility is that a snapshot of function and symptoms at this time represents the patient's new clinical baseline. For patients who continue to be functionally impaired and have refractory symptoms after 4-5 days of treatment, it may indicate progression of disease that affects prognosis.

### 4.4. Other Findings

Although it is known from previous studies [44], increased length of stay was associated with increased risk of cardiac events after discharge in this study. Longer LOS may be a marker of severity of illness, comorbidities, or other factors.

History of hypertension (HTN) in this study was found to decrease risk of cardiac events. This could be explained by the greater likelihood of having HF with preserved EF (HFPEF) in patients with longstanding HTN, but having an EF over 40% (likely HFPEF) had no effect on outcomes in this study (Table 6). It is known that lower systolic blood pressure increases risk of mortality of HF patients in community and in-hospital settings [43], but this may be a separate phenomenon from history of HTN. In a large survey of hospitalized HF patients in Europe, both a higher admission blood pressure and history of HTN were associated with increased survival at 1 year but not at 3 months [44], as was found in the present study.

### 4.5. Strengths and Limitations

The important finding of this study was that simple standard measures of function and symptoms can predict short-term outcomes in hospitalized HF patients. Strengths of this pilot study include a theoretically sound and evidence-based premise.

This pilot study has several limitations. A convenience sample was used, the sample size was small, and multivariable analysis was not completed due to the sample size. Also, a formal instrument was not used for assessing symptoms. Many well-characterized instruments exist for measuring multiple symptom dimensions, including prevalence, frequency, and severity, which might provide more insight than yes/no questions alone. While the value of the present study results lies in the simplicity of the predictive assessments, this same feature may also be a limitation. Others have noted that many risk factors can exist in the same patient, so looking at individual factors alone may not provide the most meaningful assessment of risk [43].

In addition, this study included fewer HF patients with shorter stays. Patients who were discharged within the 48-hour enrollment window were often not able to be approached for enrollment. This could mean that the study patients may have been “sicker” or more complicated to manage than their shorter-stay counterparts and therefore may not represent the full range of typical hospitalized HF patients.

Larger studies that include more patients and use formal instruments to measure multiple aspects of symptoms are needed to better characterize the association between symptoms, NYHA Classification, and short-term cardiac outcomes in hospitalized HF patients. Despite these limitations, this small pilot study provides evidence that assessments of basic HF symptoms and functional status before discharge can predict short-term patient outcomes.

## 5. Conclusion

When measured before hospital discharge, NYHA Classification and three of the most common symptoms of HF (orthopnea, fatigue, and shortness of breath) have important independent predictive value for determining risk of cardiac events within 90 days. These simple bedside assessments can be used by physicians or nurses to identify high-risk HF patients, to improve clinical decision-making in the hospital, and to provide insight for discharge planning.

Symptoms and NYHA Classification assessed after admission were not predictive of short-term outcomes in this study, underscoring the importance of the timing of assessments used for prognostication. The increased risk of cardiac events in patients with symptoms and higher NYHA Classification close to discharge suggests that symptoms and NYHA Classification could be assessed at this time and may provide outcome information when discharging patients who remain symptomatic after treatment (in this study, treatment duration was a median of 4.5 days). The various pressures to discharge patients quickly must be balanced with the goal of maximizing HF treatment and preventing negative outcomes like cardiac events, including rehospitalization.

## Figures and Tables

**Table 1:** Study procedure.

Study start—day 1	Study days 2–4	Study end—day 5 (or day of discharge)	90 days after discharge
Baseline measures	Check-in visits	Final measures	Follow-up call

Demographic: age, gender, race, marital status Clinical: vital signs, symptoms, EF Functional: home exercise (self-report), NYHA Class at home and study start, Katz Index of ADLs, KPS, SPPB Ambulation: place accelerometer monitors on ankle and thigh	Skin: check skin condition under monitors Ambulation: move accelerometer monitors to opposite ankle and thigh	Clinical: vital signs, symptoms, HRDES, EF confirmation, receipt of PT in hospital Functional: NYHA Class at study end Skin: check skin condition under monitors Ambulation: remove accelerometer monitors	Phone call to determine whether rehospitalization or emergency room visit occurred

Abbreviations: ADLs: activities of daily living; EF: ejection fraction; HRDES: high risk diagnosis for the elderly score; KPS: Karnofsky performance status scale; NYHA: New York Heart Association; PT: physical therapy; SPPB: short physical function battery.

**Table 2:** Measures of function and symptoms.

Measure	Type	Brief Description
New York Heart Association (NYHA) Classification	Function and Symptoms	Class I: no symptoms or limitations in ordinary physical activity Class II: mild symptoms and slight limitation in ordinary activity Class III: symptoms cause marked limitation even during less than ordinary activity Class IV: severe limitations; symptoms at rest

Home exercise	Function	“Do you exercise at home?”—yes/no

Katz Index of independence in activities of daily living (ADLs)	Function	Ability to perform six ADLs (bathing, dressing, toileting, transferring, continence, and feeding) independently

Short physical function battery (SPPB)	Function	Set of 3 tests (balance, gait speed, and chair stands) that objectively measure lower extremity physical function

Ambulation	Function	Direct continuous ambulation measurement via wireless accelerometers attached to lower extremity

Karnofsky performance status scale (KPS)	Function	Rating scale that assesses impact of health condition on ability to work and care for oneself

Symptom questions	Symptoms	Do you have the following symptoms—shortness of breath, fatigue, orthopnea, or lower extremity edema?—yes/no

**Table 3:** Sociodemographic and clinical characteristics of study patients (*N* = 32).

Characteristic	Value
Age, years	
Mean ± SD	58.2 ± 13.6
Range	30–92
Sex, % (*n*)	
Male	78.1 (25)
Female	21.9 (7)
Race/ethnicity % (*n*)	
Caucasian/white	59.4 (19)
African-American/black	31.3 (10)
Asian/Pacific Islander	9.4 (3)
Marital status, % (*n*)	
Married	28.1 (9)
Single	40.6 (13)
Other (widowed/divorced)	31.3 (10)
Smoking	
History of smoking, % (*n*)	75.0 (24)
Pack years, mean ± SD	21.0 ± 20.7
History of hypertension, % (*n*)	71.9 (23)
Creatinine on admission (g/dL), mean ± SD	1.9 ± 1.7
Etiology of heart failure, % (*n*)	
Ischemic	28.1 (9)
Idiopathic	65.6 (21)
Unknown/other	6.3 (2)
Ejection fraction <40%, % (*n*)	71.9 (23)
ACEi/ARB use—study end, % (*n*)	62.5 (20)
Beta blocker use—study end, % (*n*)	78.1 (25)
High-risk diagnoses for the elderly scale	
Low (0) = 9.5% chance dying in 1 year	0 (0)
Intermediate (1-2) = 31% chance	53.1 (17)
High risk (3–5) = 46% chance	31.3 (10)
Very high risk (≥6) = 74% chance	15.6 (5)
Length of hospital stay, days	
Mean ± SD	9.5 ± 9.9
Median	6.5
Range	1–41
Physical therapy—in hospital, % (*n*)	34.4 (11)
LVAD received during study admission, % (*n*)	6.3 (2)
Discharged with physical therapy, occupational therapy, or home health, % (*n*)	40.6 (13)
Cardiac events—90 days after discharge, % (*n*)	
Cardiac readmission	34.4 (11)
Heart transplant	3.1 (1)
Mortality	9.4 (3)

Abbreviations: ACEi: angiotensin converting enzyme inhibitor; ARB: angiotensin receptor blocker; LVAD: left ventricular assistive device.

**Table 4:** Functional status of study patients.

Characteristic	Value^a^		
NYHA Classification	At home	Study start^b^	Study end^c^
Class I	6.3 (2)	6.3 (2)	15.6 (5)
Class II	15.6 (5)	18.8 (6)	50.0 (16)
Class III	31.3 (10)	56.3 (18)	31.3 (10)
Class IV	46.9 (15)	18.8 (6)	3.1 (1)
Home exercise, patient reported, % (*n*)	62.5 (20)		
Katz Index of independence in ADLs, mean ± SD range 0–6	5.6 ± 1.1		
Short physical function battery—study start, mean ± SD (*n* = 31) range 0–12	6.4 ± 3.1		
Score 10–12	22.6 (7)		
Score 7–9	25.8 (8)		
Score 4–6	38.7 (12)		
Score 0–3	12.9 (4)		
Ambulation, mean ± SD			
Average time spent in each position in hospital every 24 hours			
Standing or walking	59 ± 43 minutes		
Sitting	5.5 ± 3.0 hours		
Lying	16.8 ± 3.2 hours		
Karnofsky Performance Status Scale (mean ± SD; range)	71.1 ± 9.0 (50–90)		
Score 80–100	28.1 (9)		
Score 50–79	71.9 (23)		
Score <50	0 (0)		

^a^ *n* = 32 except where indicated.

^b^Study start was 1.0 days (median) after hospital admission.

^c^Study end was 1.0 days (median) before discharge.

**Table 5:** Number of symptoms reported at beginning and end of study.

Statistic	Study start^a^	Study end^b^
*N*	32	31
Range	1–4	0–4
mean ± SD	3.3 ± 1.0	2.2 ± 1.2
Median	4	2

^a^Study start was 1.0 days (median) after admission.

^b^Study end was 1.0 days (median) before discharge.

**Table 6:** Cox univariate predictors of cardiac events within 90 days after discharge.

Variable	Hazard ratio (HR)	95% confidence interval (CI)	*P* value^a^
Age	0.974	0.93–1.02	0.266
Gender	1.548	0.34–6.99	0.570
ACEi or ARB therapy at end of study^b^	0.941	0.31–2.88	0.916
Beta blocker therapy at end of study	0.510	0.16–1.66	0.264
Ambulation—average daily time spent standing or walking in hospital	1.006	0.99–1.02	0.394
Ambulation—average daily time spent lying down in hospital	1.067	0.90–1.27	0.468
Creatinine	1.024	0.78–1.35	0.869
Home exercise, patient reported	0.667	0.22–1.99	0.467
Physical therapy in hospital	1.375	0.45–4.21	0.576
Discharged with physical or occupational therapy, or home health	1.327	0.45–3.96	0.612
Ejection fraction <40%	2.327	0.52–10.52	0.272
History of hypertension	**0.238**	**0.08**–**0.71**	**0.010**
History of smoking	1.109	0.31–4.03	0.875
Current smoking	1.484	0.70–3.15	0.304
Pack years	0.999	0.97–1.03	0.941
Karnofsky performance status scale category (KPS)	0.620	0.36–1.06	0.082
Katz Index of ADLs—study start	1.084	0.63–1.87	0.770
Length of stay	**1.085**	**1.03**–**1.15**	**0.007**
NYHA Class—home	1.607	0.79–3.25	0.188
NYHA Class—study start	1.28	0.63–2.59	0.493
NYHA Class—study end	**3.404**	**1.37**–**8.46**	**0.008**
Shortness of breath—study start^c^	24.449	0.15–39302.29	0.396
Shortness of breath—study end	**3.962**	**1.19**–**13.22**	**0.025**
Fatigue—study start	22.814	0.00–169995.56	0.492
Fatigue—study end	**9.661**	**1.24**–**75.06**	**0.030**
Orthopnea—study start	2.462	0.68–8.96	0.172
Orthopnea—study end	**12.807**	**1.65**–**99.73**	**0.015**
Edema—study start	0.649	0.20–2.11	0.472
Edema—study end	0.660	0.21–2.05	0.471
Total number of symptoms—study start	1.500	0.748–3.007	0.254
Total number of symptoms—study end	**2.341**	**1.310**–**4.182**	**0.004**
SPPB total score—study start	1.042	0.89–1.23	0.618
SPPB balance score—study start	1.473	0.84–2.57	0.173
SPPB gait score—study start	1.153	0.77–1.72	0.488
SPPB chair stand score—study start	0.869	0.57–1.33	0.515

^a^Bold indicates results with *P* < 0.05.

^b^Study end was 1.0 days (median) before discharge.

^c^Study start was 1.0 days (median) after admission.
